# Reward-Dependent Selection of Feedback Gains Impacts Rapid Motor Decisions

**DOI:** 10.1523/ENEURO.0439-21.2022

**Published:** 2022-03-28

**Authors:** Antoine De Comite, Frédéric Crevecoeur, Philippe Lefèvre

**Affiliations:** Institute of Information and Communication Technologies, Electronics and Applied mathematics (ICTEAM), Institute of Neuroscience (IoNS), Université catholique de Louvain, 1348 Louvain, Belgium

**Keywords:** decision making, perturbations, reaching movements, reward, vigor

## Abstract

Target reward influences motor planning strategies through modulation of movement vigor. Considering current theories of sensorimotor control suggesting that movement planning consists in selecting a goal-directed control strategy, we sought to investigate the influence of reward on feedback control. Here, we explored this question in three human reaching experiments. First, we altered the explicit reward associated with the goal target and found an overall increase in feedback gains for higher target rewards, highlighted by larger velocities, feedback responses to external loads, and background muscle activity. Then, we investigated whether the differences in target rewards across multiple goals impacted rapid motor decisions during movement. We observed idiosyncratic switching strategies dependent on both target rewards and, surprisingly, the feedback gains at perturbation onset: the more vigorous movements were less likely to switch to a new goal following perturbations. To gain further insight into a causal influence of the feedback gains on rapid motor decisions, we demonstrated that biasing the baseline activity and reflex gains by means of a background load evoked a larger proportion of target switches in the direction opposite to the background load associated with lower muscle activity. Together, our results demonstrate an impact of target reward on feedback control and highlight the competition between movement vigor and flexibility.

## Significance Statement

Humans can modulate their movement vigor based on the expected reward. However, a potential influence of reward on control strategies has not been documented. Here, we investigated reaching control strategies in different contexts associated with explicit rewards for one or multiple goals, while exposed to external perturbations. We report two strategies: reward could either increase feedback gains, or promote flexible switches between goals. The engagement of peripheral circuits in the modulation of feedback gains was confirmed by the application of a background load that biased feedback vigor directionally, evoking differences in switching behavior in the opposite direction. We conclude that feedback vigor and flexible changes in goal are two competing mechanisms to be selected when interacting with a dynamic environment.

## Introduction

From the toddler picking their favorite toys to the footballer selecting the best path through opponents, humans manifest the exquisite ability to plan and select movements. Movement planning is the process that integrates many task-related factors to select the best control strategy for the task (Wong et al., 2015). Amongst these numerous factors, the reward associated with the task induces a modulation of movement vigor in saccadic eye movements (Manohar et al., 2015, 2017) and upper limb reaching movements (Esteves et al., 2016; Summerside et al., 2018). Moreover, recent studies reported that higher reward increases visuomotor responses to disturbances (Carroll et al., 2019) and that the increase of vigor associated with reward is correlated with a reduction of movement variability and an increase in co-contraction (Codol et al., 2020). Together, these previous results suggested an influence of reward on movement planning strategies.

Besides this impact on movement planning, reward also has an influence on movement selection. Indeed, the selection of the best alternative between different options is biased toward movements associated with the highest reward (Trommershäuser et al., 2003, 2008). Similarly, when humans have to select a target, their choice is biased by parameters such as the biomechanical costs incurred when reaching to each potential option, resulting in target selection toward less effortful movements (Cos et al., 2011; Morel et al., 2017).

The commitment to an action actually results from a distributed consensus between low level sensorimotor representations of movement costs (e.g., motor costs) and high level cognitive representations of their outcomes (e.g., reward; Cisek, 2012). Here, we explored the impact of target reward on fast feedback control strategies and tested the distributed consensus theory in a dynamical context by probing the effect of movement reward on feedback control and online motor decisions. Recent studies have sought to investigate whether and how much the factors that characterize action selection during movement planning could also influence movement selection when the hand has already started moving. A first body of work have shown that dynamical changes in target selection can be triggered by mechanical (Nashed et al., 2014) or visual (Kurtzer et al., 2020; Michalski et al., 2020) perturbations occurring during movement. More recently, some studies demonstrated that cognitive factors, such as the reward distribution of a redundant target, also influence online motor decisions (Marti-Marca et al., 2020; Cos et al., 2021). They revealed that the reward distribution of a redundant target influences online motor decisions and suggested a link between the state of the limb (position and speed) at perturbation onset and the outcome of the decision. However, whether the reward of competing alternatives or the level of muscle activity could influence online motor decisions has not been explored yet.

In the present work, we addressed the relationship between target reward and feedback control as well as online motor decisions by applying perturbations while participants performed reaching movements toward one or several targets that differed explicitly by their associated rewards. We hypothesized that the influence of reward on movement planning was linked to the selection of feedback gains, which could impact one’s ability to flexibly change target during movement. In a first experiment, we investigated the influence of reward on feedback control strategies. We then investigated the impact of reward on feedback control when participants had the opportunity to reach to different goals. The goal of the third experiment was to study the competition between feedback gains and the ability to flexibly change movement goal during movement.

We first reproduced previous findings of reward-related increase in velocity toward the target. Importantly, we uncovered that this modulation was associated with an increase in feedback gains and muscle activity. In a second experiment, we observed that the difference in reward between alternative goals could bias online motor decisions and, interestingly, found out that the overall increase in movement vigor was negatively correlated with the potential selection of a new target. Our third experiment confirmed that biases in feedback gains induced experimentally were negatively correlated with the ability to switch goal during movement. These findings demonstrate that movement reward modulates both planning and feedback control, and involves the peripheral motor system through modulation of muscle co-contraction and reflex gains. Moreover, we highlight that this modulation was detrimental to the ability to flexibly switch to a new goal during movement.

## Materials and Methods

### Participants

A total of 53 participants were enrolled in this study and took part to one of the three experiments. The first group performed experiment 1 and included 14 right-handed participants (seven females) ranging in age from 21 to 27. The second group performed experiment 2 and included 20 right-handed participants (14 females) ranging in age from 20 to 46. The last group performed experiment 3 and included 19 right-handed participants (11 females) ranging in age from 18 to 52. Participants were naive to the purpose of the experiments and had no known neurologic disorder. The ethics committee of the host institution approved the experimental procedures and participants provided their written informed consent before the experiment.

### Experiments

For the three experiments, participants sat on an adjustable chair in front of a Kinarm end-point robotic device (KINARM) and grasped the handle of the right robotic arm with their right hand. The robotic arm allowed movements in the horizontal plane and direct vision of both the hand and the robotic arm was blocked. Participants were seated such that at rest their arm was vertical and their elbow formed an angle of ∼90°. Their arm was unconstrained and their forehead rested on a soft cushion attached to the frame of the setup. A virtual reality display placed above the handle allowed the participants to interact with virtual targets. A white dot of 0.5-cm radius corresponding to the position of the handle was shown on this display during the whole experiment.

### Experiment 1

**Figure 1.**
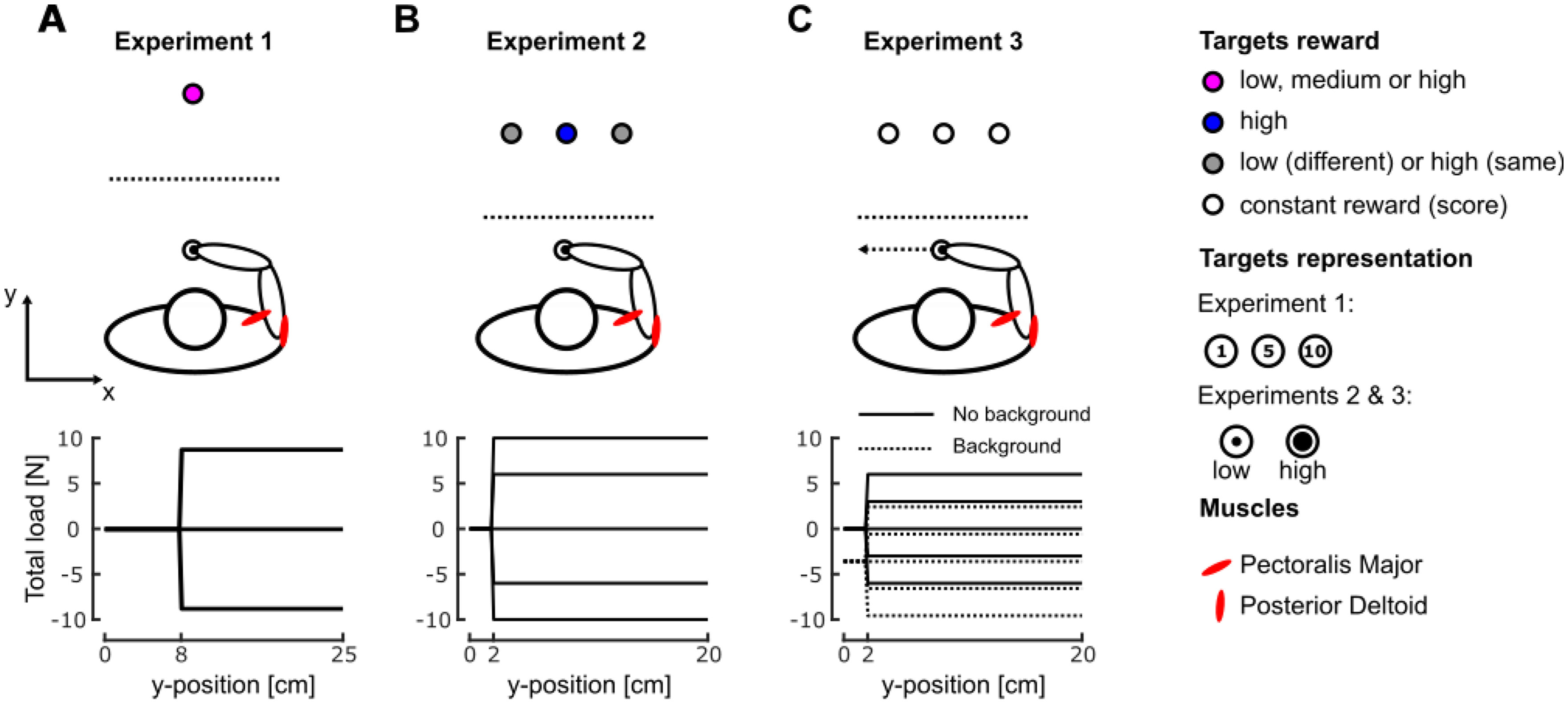
Task paradigms. ***A***, Representation of the task paradigm of experiment 1. Participants controlled a hand-aligned cursor represented by the black dot on a virtual reality display. They had to reach for the goal target, represented by the magenta goal target in front of them. This goal target could have a low, medium, or high reward (1, 5, or 10 points). The bottom part of the panel represents the load profiles that participants could experience. ***B***, Representation of the task paradigm of experiment 2. Participants had to reach for any of the three targets presented in front of them. The central target always had a high reward whereas the two others either had a low or a high reward. The bottom part of the panel represents the load profiles that participants could encounter during movements. ***C***, Representation of the task paradigm of experiment 3. Participants had to reach for any of the three targets presented in front of them. During the second half of the trials, a background load force directed leftward was applied prior and during the movement (dashed line bottom panel). The bottom part of the panel represents the possible profiles of the total load forces (perturbation load + background load). EMG data from PM and PD were collected during all experiments.

In experiment 1 (Fig. 1*A*, top), participants (*N* = 14) were instructed to perform reaching movements to a small circular goal target (1.5-cm radius) located at 25 cm in the *y*-direction from the start position, a red disk of 1.5-cm radius. Participants had first to put the hand-aligned cursor in the start position, which turned green as they reached it. After a random time delay (drawn from an uniform distribution between 1 and 2 s), the goal target appeared as a red disk containing a number (1, 5, or 10) that corresponded to the reward participants would receive if they reached and stabilized within the target for a prescribed time window. Reaction time was not constrained and participants could start the movement whenever they wanted (mean reaction time 518 ms). Following the exit of the start position, participants had up to 600 ms to reach the goal target and keep the cursor inside for at least 500 ms. The goal target turned green at the end of successful trials, or remained red otherwise. During movements, a mechanical perturbation load could be applied to participants’ hand (33% of the trials). This load consisted of a lateral step force of ±9 N, with a 10-ms linear build-up, aligned with the *x*-axis. This force was triggered when the hand-aligned cursor crossed a virtual line located at 8 cm from the center of the start position (Fig. 1*A*, bottom). Unperturbed and perturbed trials as well as trials with different rewards were randomly interleaved such that participants could not predict the occurrence or the direction of the perturbations. Participants started with a 27-trial training block to become familiar with the task and the force intensity of perturbation loads. After completing this training block, they performed six blocks of 72 trials interleaved with pauses of 3–5 min to prevent muscle fatigue. Each 72-trial block included: 48 unperturbed trials (16 with each target reward) and 24 trials which contained mechanical perturbations (leftward or rightward, eight of each reward condition). Participants performed a total of 432 trials, including 24 for each perturbed condition (direction of the mechanical perturbation and value of the target reward). A total score corresponding to the cumulative sum of individual movement rewards was projected next to the goal target. Participants were compensated for their participation according to a conversion of this total score. This conversion was calculated such that each participant received between 10 and 15 euros as an incentive to score a maximum number of points during the experiment.

### Experiment 2

Experiment 2 was designed to assess the effect of reward on online motor decisions between competing motor goals. Instead of reaching to a single target, participants (*N* = 20) were instructed to perform reaching movements to any of three circular targets (1.5-cm radius) located at 20 cm in the *y*-direction from the same start position as in experiment 1 (Fig. 1*B*, top). As in experiment 1, the goal targets appeared after participants stabilized the hand-aligned cursor in the start position. All three goal targets appeared in each trial, the central one being aligned along the *y*-axis with the start position and the other two equidistant from this central target at 9 cm along the *x*-axis. These targets were presented as an inner disk of radius 0.7 or 1.2 cm inside an outer circle of radius 1.5 cm. The purpose of the inner disk was to show the reward associated with the target: the larger the diameter of this disk, the higher the reward. There were two different conditions of reward: either all the targets had the same large reward (same values condition) or only the central target had a large reward while the other two had lower rewards (different values condition). In a pilot study, we considered a third reward configuration: the central target had a small reward while the other two had higher rewards. We observed that, in this third configuration, the behavior in the absence of perturbation load was biased toward the lateral targets. We therefore decided to exclude this condition to keep the conditions in which participants spontaneously reached for the center target for the largest proportion of trials in the absence of any perturbation load. After a random time delay (drawn from a uniform distribution between 1.5 and 3 s), the inner disks of the goal targets turned white and participants had to reach any of these within 400–1000 ms to pass the trial. Similar to experiment 1, the reaction time was not constrained (mean reaction time 311 ms). The trial was successfully completed if participants reached any goal target in the prescribed time window and stabilized the cursor in it for 500 ms. The inner disks of the goal targets turned green if the trial was successful and red otherwise. As in experiment 1, a mechanical perturbation load could be applied to participant’s hand (50% of the trials, ±6 or ±10 N, 10-ms build-up aligned with the *x*-axis; Fig. 1*B*, bottom). This perturbation was triggered when the hand-aligned cursor crossed a virtual line located at 2 cm from the start position. Unperturbed and perturbed trials as well as trials with different reward distributions and force intensities were randomly interleaved. Participants started with a 58-trial training block followed by six blocks of 80 trials. Pauses of 3–5 min were introduced between blocks to prevent muscle fatigue. Each 80-trial block included: 40 unperturbed trials and 40 trials which contained mechanical perturbations. Participants performed a total of 480 trials including 30 trials of each perturbation condition (reward condition and mechanical perturbation condition). Participants were compensated for their participation using the same conversion rule as in experiment 1.

### Experiment 3

The third experiment was a variant of experiment 2 and was designed to test the possible impact of muscle activity on online motor decisions by applying a background force orthogonally to the reach path (Fig. 1*C*, top). Participants had to perform reaching movements to any of the three targets, located as in experiment 2. These targets were identical to the large reward target of experiment 2 and the time course of events in the trial was similar (mean reaction time 424 ms) as well except that a leftward background mechanical load of 4 N was applied as participants reached the start position and remained on throughout the trials. As in the experiment 2, a mechanical perturbation load could be applied to participant’s hand during movement (33% of the trials). This load consisted of a ±3 or ±6 N with a 10-ms build-up triggered when the hand-aligned cursor crossed a line located at 2 cm from the start position (Fig. 1*C*, bottom) and was added to the background load. Participants first performed a 21-trial training block which did not involve background load. After completing this training, participants performed four blocks of 60 trials which did not include the background load. Each 60-trial block included 40 unperturbed trials and 20 trials with mechanical perturbations and they were interleaved with pauses of 3–5 min. After these 60-trial blocks, participants performed a second 21-trial training block which included the background load. Once this second training block was completed, participants performed a second set of four blocks of 60 trials which included the background load. They thus performed a total of 480 trials among which 24 of each condition (with different perturbation loads and background load on or off). To motivate participants, a score corresponding to their number of successful trials was projected next to the goal targets. Participants were compensated a fixed amount for their participation.

### Data collection and analysis

Raw kinematics data were sampled at 1 kHz and low-pass filtered using a fourth order double-pass Butterworth filter with cutoff frequency of 20 Hz. Hand velocity along the *y*-axis was computed from numerical differentiation of the position data using a fourth order centered finite difference.

Surface EMG electrodes (Bagnoli surface EMG sensor, Delsys Inc) were used to record muscles activity during movements. We measured the pectoralis major (PM) and the posterior deltoid (PD) based on previous studies (Crevecoeur et al., 2019; De Comite et al., 2021) that showed that in this configuration they are stretched by the application of forces opposite to their action, and therefore largely recruited by the feedback responses. Before applying the electrodes, the skin of the participant was cleaned and abraded with cotton wool and alcohol. Conduction gel was applied on the electrodes to improve the quality of the signals. The EMG data were sampled at a frequency of 1 kHz and amplified by a factor of 1000. A reference electrode was attached to the right ankle of the participant. Raw EMG data from the PM and PD were bandpass filtered using a fourth order double-pass Butterworth filter (cut-offs: 20 and 250 Hz), rectified, aligned to force onset and averaged across trials or time windows as specified in Results. The time windows selected for the temporal averaging are the short-latency (20–50 ms), the long-latency (50–100 ms), and the voluntary time epochs (100–180 ms) as proposed in previous work (Pruszynski et al., 2008; Pruszynski and Scott, 2012).

EMG data were normalized for each participant to the average activity collected when participants maintained postural control at the start position against a constant force of 9 N. Data from the PM were normalized by the EMG activity in the same muscle while performing postural control against a rightward force whereas data from the PD were normalized by the EMG activity in the same muscle while performing postural control against a leftward force. This calibration procedure was applied after the second and the fourth blocks in the first two experiments and after the first, third, fifth, and seventh blocks in the third experiment. Data processing and parameters extractions were performed using MATLAB 2019a.

In experiment 1, we fitted linear mixed models to determine the effect of the target reward on the kinematics and EMG activity. These models were fitted using the *fitlme* function of MATLAB and the formula used was the following:

(1) $$Parameter=\beta_{0}+\beta_{1}\text{ × }Reward+\alpha_{i}.$$

The fixed predictors were the intercept $\left(\beta_{0}\right)$ and the reward condition $\left(\beta_{1}\right)$ while the participants were included as a random offset $\left(\alpha_{i}\right)$. For all linear mixed model analyses that we performed, we reported the estimate for $\beta_{1}$, the *t* statistics for this estimate as well as the corresponding *p*-value and the $r^{2}$ of the model. One-tailed paired *t* tests were used for *post hoc* analyses where we collapsed data across trials and participants to compare the different conditions. Effect size for these tests were reported using Cohen’s *d* defined as the difference between the means of the two populations divided by the standard deviation of the whole sample.

To analyze the data from experiments 2 and 3, we designed a multilinear logistic regression model to infer the effect of reward distribution and background load on target choice as the dependent variable, respectively. Considering that the dependent variable was a discrete variable (the chosen target), we use the following logistic regression model:

(2) $$\log\left(\frac{P\left(Lateral\;target\right)}{P\left(Central\;target\right)}\right)=\beta_{0}+\beta_{1}\text{ × }Parameter_{1}+\beta_{2}\text{ × }Parameter_{2},$$

where the first effect ( $\beta_{1}$) was the reward condition (experiment 2) or the presence of a background load (experiment 3) and the second effect $\left(\beta_{2}\right)$ was the intensity of the perturbation load. For these logistic regressions, we reported the estimates for $\beta_{1}$ and $\beta_{2}$, their corresponding *t* statistics as well as their *p*-value. For *post hoc* analyses in experiment 2, we used a one-tailed Wilcoxon signed ranked test for which we reported the ranksum, the *z* statistics when provided, the *p*-value as well as the effect size given by the Cohen’s *d* as defined above. In order to investigate the asymmetry in the parameters $\beta_{1}$ obtained in experiment 3, we used bootstrap resampling on the individual data to generate 1000 estimates of the $\beta_{1}$ parameter for each condition (leftward perturbation vs rightward perturbation) using the multilinear logistic regression described above. We then assessed the asymmetry of the effect by investigating whether the 95% confidence interval of the difference between these two $\beta_{1}$ parameters contains 0 (Efron, 1979).

In order to determine the effect of the background load on the baseline muscle activity in experiment 3, we fitted a linear mixed model with interaction terms following this equation:

(3) $$EMG=\beta_{1}\text{ × }Background+\beta_{2}\text{ × }Muscle+\beta_{12}\text{ × }Muscle:Background+\alpha_{i}.$$

Where the first term $\left(\beta_{1}\right)$ refers to the background condition, the second ( $\beta_{2}$) to the muscle, the third one ( $\beta_{12}$) to the interaction term and the last one ( $\alpha_{i}$) to the random offset of participants. For all these β, we reported their estimated value as well as their *t* statistics, associated *p*-value, and the $r^{2}$ of the model. Significance was considered at the level of *p* = 0.05 although we decide to exactly report any *p*-value that was larger than *p* = 0.005 as previously proposed (Benjamin et al., 2018). In the figures, we reported significant differences for the level *p* < 0.05 (*), *p* < 0.01 (**), and *p* < 0.005 (***).

## Results

### Influence of the target reward on feedback corrections during movement

To determine whether target reward influences feedback corrections during movement, participants were instructed to perform reaching movements to a goal target associated with a reward that could change across trials (see Materials and Methods). During movements, mechanical perturbation loads could be applied to reveal feedback corrections. The occurrence of feedback corrections was assessed by looking at movement kinematics and EMG responses of the muscles stretched by the perturbations.

**Figure 2.**
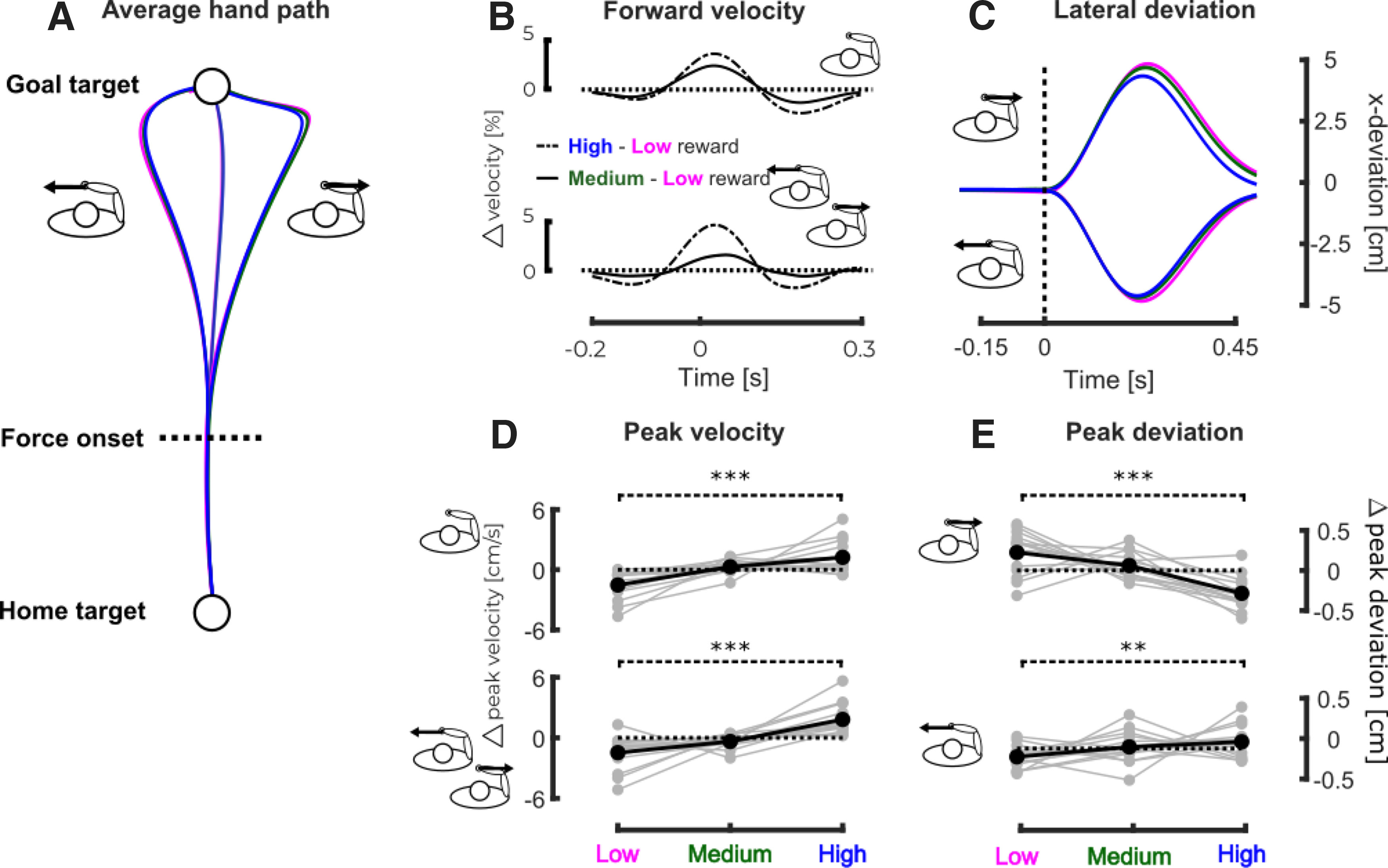
Experiment 1, kinematics. ***A***, Mean hand path across participants for the different conditions of the first experiment. The magenta, green, and blue traces, respectively, correspond to the low, medium and high reward conditions. The dashed line represents the onset of the mechanical perturbation. ***B***, Mean difference in forward velocity between the high and low (dash-dot line) and medium and low (full line) for the unperturbed (top) and perturbed (bottom) trials. The time axis is aligned on the force onset. ***C***, Mean hand deviation across participants for the perturbed trials. The hand deviation has been obtained by subtracting the mean hand path to the perturbed hand path in the same reward condition for every subjects. The top part of the graph represents the trials perturbed to the right whereas the bottom part of the graph represents the trials perturbed to the left. ***D***, Group mean (black) and individual means (gray) of the differential forward peak velocity for the unperturbed trials (top) and perturbed trials (bottom) as a function of the reward condition with respect to average forward peak velocity. ***E***, Group mean (black) and individual means (gray) of the difference in hand deviation with respect to the mean hand deviation for leftward (top) and rightward (bottom) perturbation in the three reward conditions with respect to the average hand deviation; *p* < 0.05 (*), *p* < 0.01 (**), *p* < 0.005 (***).

The mean hand path trajectories across participants are represented in Figure 2*A* for the different perturbations and reward conditions. Consistent with previous work (Shadmehr et al., 2016; Summerside et al., 2018), we observed a significant increase in forward peak velocity with increasing reward values. Figure 2*B* shows the differences in the forward velocities between the high (dash-dot lines) or medium (full lines) and low reward conditions during the unperturbed (top) and perturbed (bottom) trials. The peak forward velocity (defined as the velocity component aligned with the main reaching direction) increased with increasing reward value both for unperturbed (linear mixed model: $\beta_{1}$ = 0.013, *t* = 6.51, *p* < 0.005, $r^{2}$ = 0.76; Fig. 2*D*, top) and perturbed (linear mixed model, right: $\beta_{1}$ = 0.014, *t* = 3.76, *p* < 0.005, $r^{2}$ = 0.78, left: $\beta_{1}$ = 0.018, *t* = 4.68, *p* < 0.005, $r^{2}$ = 0.79; Fig. 2*D*, bottom) trials. *Post hoc* comparisons between low and high reward conditions revealed a significant increase of peak velocity with reward for all perturbation conditions (one-tailed paired *t* tests, unperturbed: *t* = −7.48, *p* < 0.005, *d* = 0.12, left: *t* = −5.37, *p* < 0.005, *d* = 0.16 and right: *t* = −3.99, *p* < 0.005, *d* = 0.13). We did not observe any modulation of the reaction time required to initiate movement with the reward (linear mixed model *p* > 0.05) since we instructed participants to initiate movements whenever they wanted (see Materials and Methods).

The effect of the mechanical perturbation on the movement kinematics was also dependent on the reward value. Indeed, the maximum lateral hand deviation induced by the mechanical perturbation (Fig. 2*C*), computed as the difference between the hand paths in the perturbed conditions and the mean hand path in the corresponding unperturbed reward condition for each participant, depended significantly on the reward condition. For both perturbation directions (Fig. 2*E*, top for leftward and bottom for rightward perturbations), we observed a significant decrease in the maximal hand deviation along the *x*-axis with increasing reward value (linear mixed models, right: $\beta_{1}$ = −0.0025, *t* = −4.98, *p* < 0.005, $r^{2}$ = 0.38 and left: $\beta_{1}$ = −0.0009, *t* = −2.25, *p* = 0.024, $r^{2}$ = 0.35). *Post hoc* comparisons between low and high reward conditions revealed a significant decrease for both perturbation directions (one-tailed paired *t* tests, right: *t* = 5.31, *p* < 0.005, *d* = 0.14 and left: *t* = 2.34, *p* = 0.009, *d* = 0.3).

Based on these kinematics analyses and previous studies showing that faster movements and smaller hand deviations induced by perturbations are correlated with high EMG activity (Crevecoeur et al., 2019), we hypothesized that the EMG activity in PM and PD during movement scaled with increasing reward. We investigated this effect both for baseline activity measured during unperturbed trials and for feedback responses to perturbation loads.

**Figure 3.**
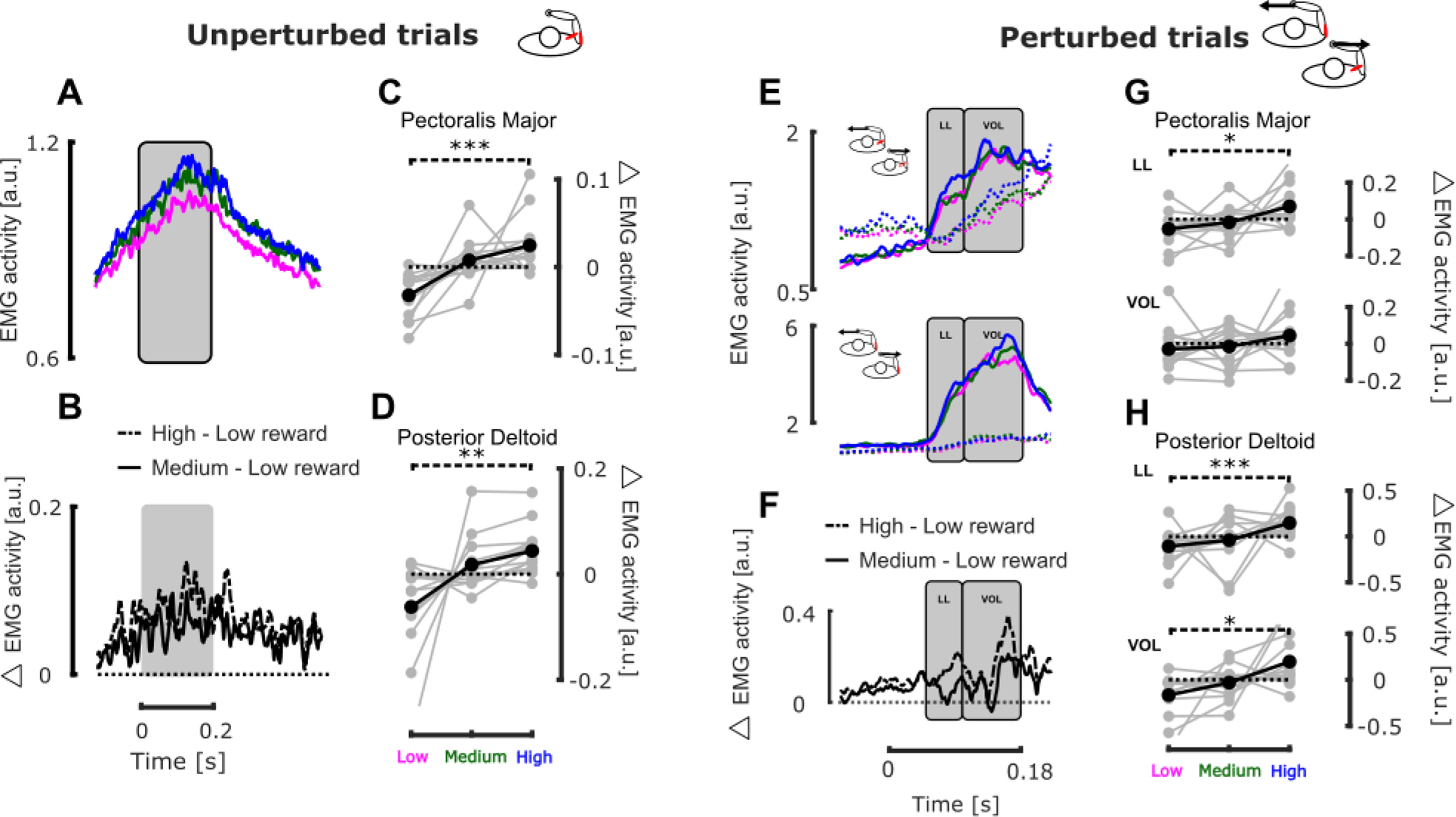
Experiment 1, EMG activity. ***A***, Mean EMG activity collapsed across muscles and participants for unperturbed trials. The time axis is aligned on force onset. ***B***, Mean differences in EMG activity collapsed across muscles and participants between high and low (dash-dot line) and medium and low (full line) reward conditions for unperturbed trials. ***C***, Group mean (black) and individual means (gray) PM EMG activity binned between 0 and 200 ms after force onset for unperturbed trials. ***D***, Group mean (black) and individual means (gray) PD EMG activity binned between 0 and 200 ms after force onset for unperturbed trials. ***E***, Group mean EMG activity in PM (top) and PD (bottom) when they were stretched (full lines) or shortened (dashed lines) by mechanical perturbations. ***F***, Mean differences in EMG activity collapsed across muscles and participants between high and low (dash-dot line) and medium and low (full line) reward conditions for agonist muscles in presence of perturbation load. ***G***, Group mean (black) and individual means (gray) differential EMG activity in PM binned in the long latency (50–100 ms, top) and voluntary epochs (100–180 ms, bottom) as a function of the reward condition. ***H***, Group mean (black) and individual means (gray) of the differential EMG activity in PD binned in the long latency (50–100 ms, top) and voluntary epochs (100–180 ms, bottom) as a function of the reward condition; *p* < 0.05 (*), *p* < 0.01 (**), *p* < 0.005 (***).

We observed a positive correlation between the EMG activity during unperturbed trials and the value of the target reward. Figure 3*A* represents the mean EMG activity collapsed across muscles and participants for unperturbed trials while the differences between these collapsed EMG activities in the high (dash-dot line) or medium (full line) and the low reward condition are represented in Figure 3*B*. We binned the EMG activity of each trial in a time bin ranging from 0 to 200 ms after perturbation onset (Fig. 3*A*,*B*, gray rectangle) and fitted a linear mixed model (see Materials and Methods) on these binned values to determine whether reward had an influence on the EMG activity (deviations from the mean binned EMG activity in the different reward conditions are represented in Fig. 3*C*,*D* for PM and PD, respectively). We observed an increase in EMG activity with the reward in both muscles (PM: $\beta_{1}$ = 0.028, *t* = 4.603, *p* < 0.005, $r^{2}=0.68$, PD: $\beta_{1}$= 0.053, *t* = 5.98, *p* < 0.005, $r^{2}$ = 0.66). *Post hoc* analyses performed on individual data showed that EMG activity was larger in the high reward condition than in the small one for both muscles (one-tailed paired *t* tests: pectoralis, *t* = 4.14, *p* < 0.005, *d* = −0.118 and deltoid, *t* = −2.92, *p* = 0.0059, *d* = 0.1653).

The EMG response to mechanical perturbation in the agonist muscles was also modulated by the reward value. Indeed, linear mixed model analyses performed on the responses measured in PM and PD, when, respectively, a rightward or leftward perturbation occurred, showed a significant increase of EMG activity with increasing reward in the long-latency epochs (50–100 ms). We reported the EMG activities collapsed across muscles and participants in Figure 3*E* as well as the difference in these activities between the high (dash-dot line) or medium (full line) and the low reward condition in Figure 3*F*. For each perturbation direction, we binned the EMG activity of the stretched muscle in the long latency (LL 50–100 ms after force onset) and voluntary (VOL 100–180 ms after force onset) epochs. Figure 3*G*,*H*, respectively, represents the deviation from the mean binned EMG activity in these two time bins (LL top and VOL bottom) for PM and PD in the different reward conditions. In PM, we observed a significant increase in the LL window (mixed model: $\beta_{1}$ = 0.0615, *t* = 2.89, *p* < 0.005, $r^{2}$ = 0.64), but although a positive tendency emerged in the VOL window, no significant increase was observed (mixed model: $\beta_{1}$ = 0.036, *t* = 1.616, *p* = 0.106, $r^{2}$ = 0.69). Individual pairwise *post hoc* comparisons between low and high conditions confirmed these findings (one-tailed paired *t* tests: LL, *t* = −2.48, *p* = 0.0137, *d* = 0.1592 and VOL, *t* = −1.18, *p* = 0.128, *d* = 0.08). The same holds for PD in which we found a significant increase of EMG activity in LL window with the reward (mixed model: $\beta_{1}$ = 0.216, *t* = 2.12, *p* = 0.034, $r^{2}$ = 0.63) but no significant effect in the VOL window (mixed model: $\beta_{1}$ = 0.181, *t* = 1.887, *p* = 0.059, $r^{2}$ = 0.77). In this case, however, the individual pairwise comparisons between low and high conditions revealed a significant increase in both time windows (one-tailed paired *t* tests: LL, *t* = −3.68, *p* < 0.005, *d* = 0.11 and VOL, *t* = −2.57, *p* < 0.01, *d* = 0.07).

An interesting question is whether the effect of target reward reported here could only be attributable to higher movement speed. In other words, could it be that the impact of a higher reward is an increase in movement speed that would therefore modulate the behavior. To answer that question, we compared the lateral hand deviation observed in trials that have similar peak velocity and investigated whether, in these trials, the reward condition modulates the lateral hand deviation. We performed a linear mixed model analysis on the absolute values of these lateral hand deviations and reported an effect of the reward condition: smaller deviations for higher reward value ( $\beta_{1}$ = −0.0011, *t* = −2.61, and *p* = 0.009). This result confirms that reward does not only modulate movement vigor but also the feedback responses to mechanical perturbations.

Therefore, the results of experiment 1 revealed that the value of the target reward influenced both the movement kinematics and the EMG activity recorded during movement. Indeed, we showed that the hand deviation induced by mechanical perturbations decreased with increasing reward value for both rightward and leftward perturbations. Moreover, the forward peak velocity of reaching movement increased with increasing reward value. Finally, EMG activity in both PM and PD increased with increasing reward value for unperturbed trials and in the long-latency response window for perturbed movement when the muscles were stretched by the perturbation. The modulation of forward hand velocity and baseline EMG activity which also produced increases in feedback responses to perturbation loads was consistent with an increase in control gains previously observed in uncertain dynamical contexts, which was interpreted as a robust control strategy (Crevecoeur et al., 2019).

### Influence of the reward of the different options on online motor decisions

In experiment 1, we showed that reward of the goal target had an influence on the way humans perform reaching movements to this target similarly to other task parameters such as target shape, presence of obstacles, etc. Moreover, previous studies have shown that these task parameters that modify the control strategies could also influence online motor decisions (Nashed et al., 2014). We therefore designed a second experiment to determine whether reward could also influence online motor decisions. In this second experiment, participants had to reach to any of three potential targets aligned orthogonally to the main reaching direction (see Materials and Methods). The central target always had a large reward while the two lateral targets could either have lower reward or a reward equal to that of the central target. We assessed the effect of the difference between central and lateral rewards on online motor decisions by investigating the frequencies of reaching for the lateral targets. Mechanical perturbations that could occur during movement were used to evoke changes in goal target. Because perturbations were unpredictable, a change in reaching frequency for the lateral targets dependent on the perturbation load was indicative of a perturbation-mediated change in goal that occurred during movement. The biomechanical and EMG states at perturbation onset in the same condition were also investigated to determine whether they had an influence on the future decision.

**Figure 4.**
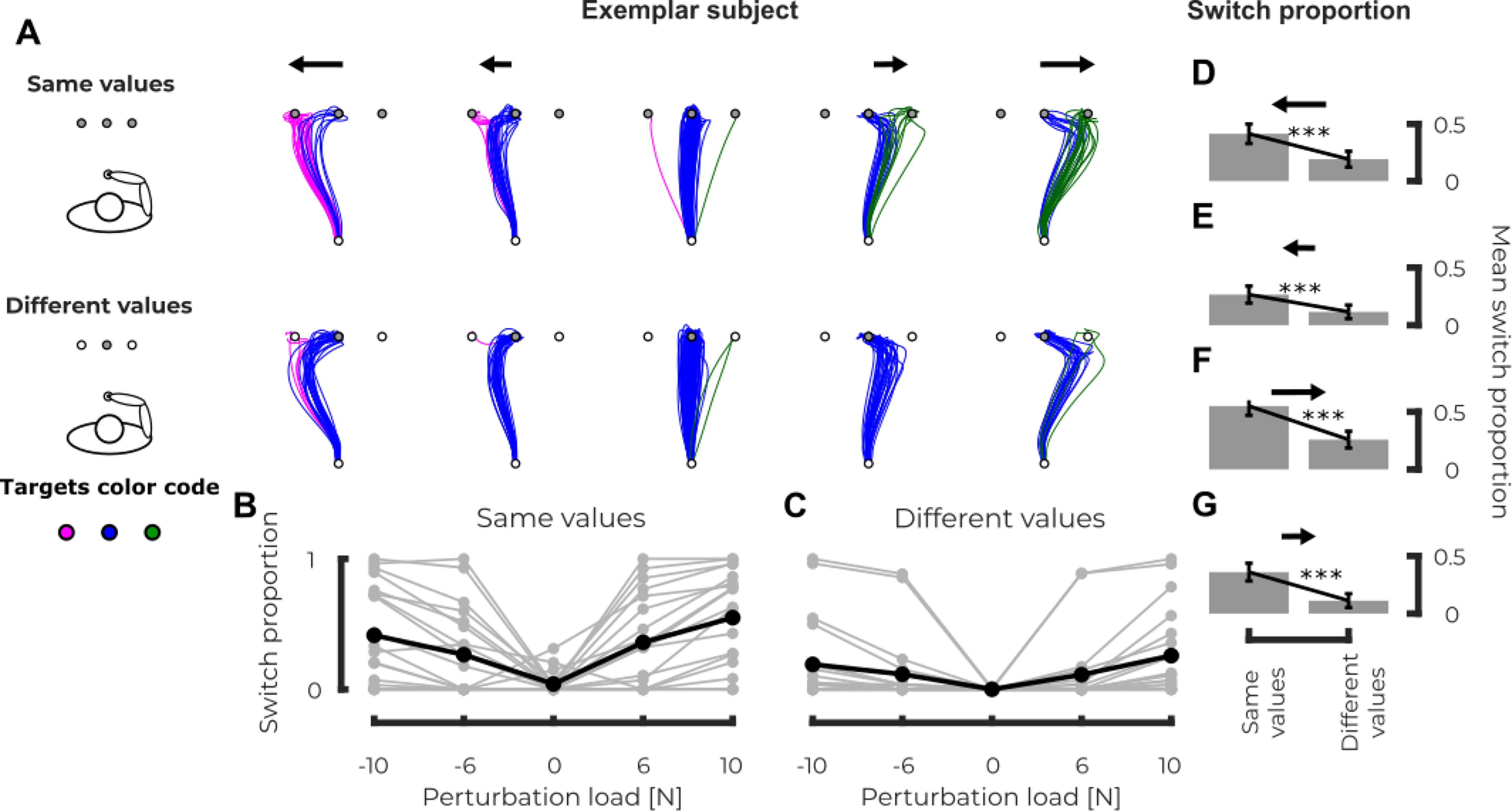
Experiment 2, kinematics. ***A***, Representation of hand path of individual trials for a representative subject in the same condition on top (all the targets had the same reward) and in the different condition on bottom (the central target had a higher reward than the other two). The different columns represent the different force levels (from right to left, large leftward perturbation to large rightward perturbation). Magenta, blue, and green paths, respectively, represent the paths that reached the left, central, and right targets. ***B***, Group mean (black) and individual means (gray) of the switch proportion (i.e., fraction of trials that reached either the left or right targets) as a function of the applied load for the same condition. ***C***, Group mean (black) and individual means (gray) of the switch proportion (i.e., fraction of trials that reached either the left or right targets) as a function of the applied load for the different condition. ***D–G***, Comparison of the switch proportion for the same (left) and different (right) conditions for the trials with large leftward force, small leftward force, small rightward force and large rightward force, respectively; *p* < 0.05 (*), *p* < 0.01 (**), *p* < 0.005 (***).

First, we observed that the reward of the lateral targets had a clear effect on the frequency of trials that ended on these targets. Figure 4*A* represents the hand paths of a typical participant toward the different targets in various conditions. In general, in the absence of perturbation, subjects tend to reach to the central target except for some trials (<1% in the different values condition and 8% in the same values condition). In all cases, the frequency of lateral target increased with the magnitude of the perturbation (Fig. 4*A*, top and bottom for same and different values conditions, respectively). In addition, there was a significant effect of the lateral targets reward on the frequency of lateral target reach: lower frequencies for different rewards. In order to determine the significance of these effects, we identified for each trial the target that was reached at the end of the movement and fitted a multilinear logistic regression on these data to determine whether the reward condition and the force had an influence on the target reached (see Materials and Methods). We observed a significant effect of the reward for both the left ( $\beta_{1}=1.103,t=10.84,p<0.005$) and right ( $\beta_{1}=1.666,t=18.45,p<0.005$) targets versus the central one. These positive values indicate that the reach proportion to the lateral targets is larger in the same than in the different condition (see Fig. 4*B*,*C* for the same values and different values conditions, respectively). The intensity of the perturbation loads also had a significant effect for both lateral targets versus the central one (left: $\beta_{1}$ = −1.33, *t* = −26.82, *p* < 0.005 and right: $\beta_{1}$ = 1.25, *t* = 29.07; *p* < 0.005). Because of the sign of the force in the regression model, in both cases the frequency of lateral target reach increased with the force magnitude in absolute value. *Post hoc* analyses performed at fixed force levels showed a significant effect of the reward condition on the reaching proportion for all the perturbed conditions. We observed a smaller reach proportion to the left target in the different values condition compared with the *same values* condition for both perturbation directions [one tailed Wilcoxon signed-rank test: ranksum = 3, *p* < 0.005, *d* = 0.61 (Fig. 4*D*) and ranksum = 1, *p* < 0.005, *d* = 0.49 (Fig. 4*E*) for loads of −10 and −6 N, respectively]. The mirror effect was observed for the right target: a decrease in the reach proportion in the different values condition for both perturbation directions [one-tailed Wilcoxon signed-rank test: ranksum = 0, *p* < 0.005, *d* = 0.74 (Fig. 4*F*) and ranksum = 3, *p* < 0.005, *d* = 0.78 (Fig. 4*G*) for loads of 6 and 10 N, respectively].

These results showed that participants took the reward distribution of the options offered by the three targets into account while deciding which target they should reach. The next question that we will address is whether any parameters linked to the current state of the limb could modify the decision between the different motor outcomes.

**Figure 5.**
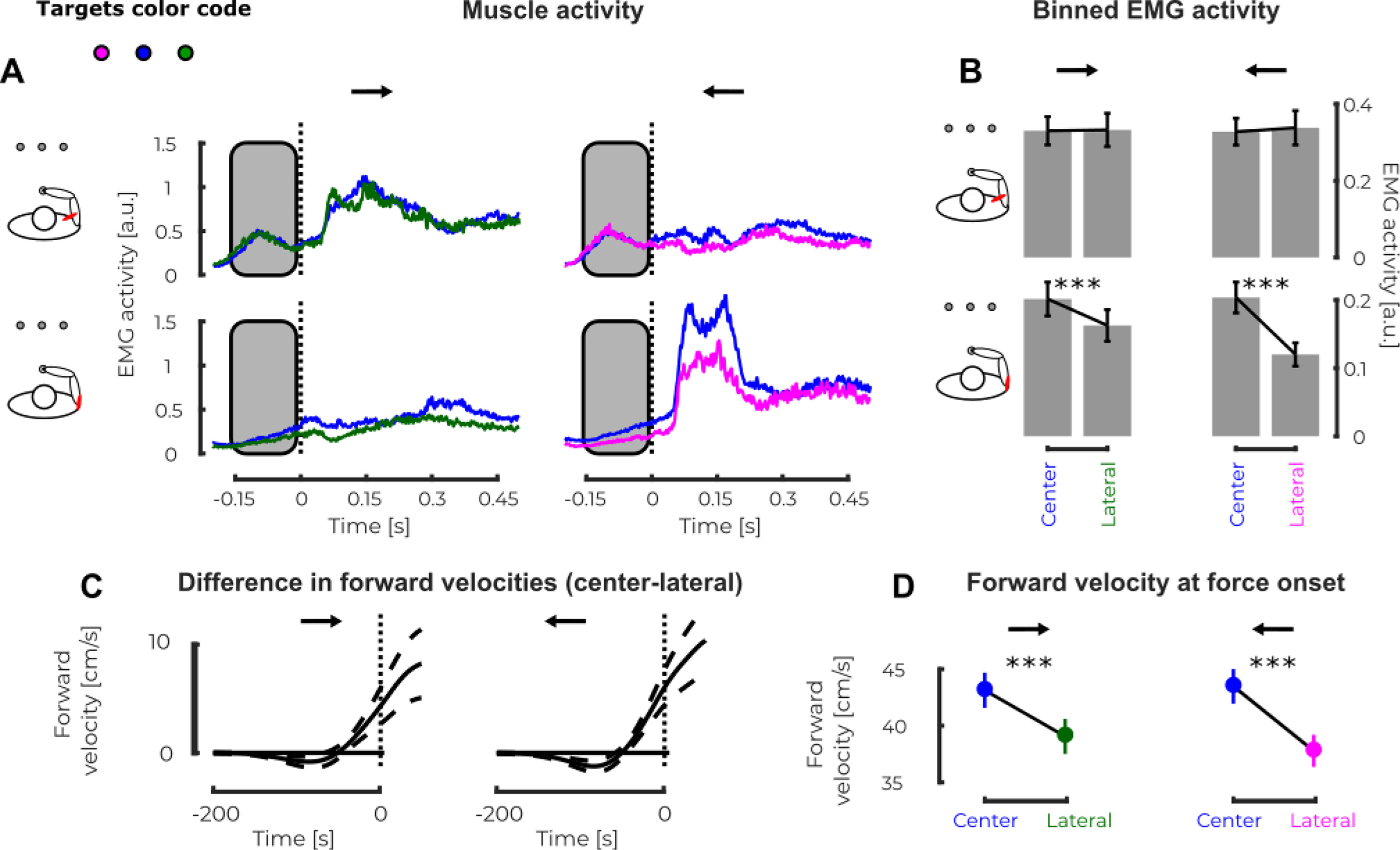
Experiment 2, EMG activity. ***A***, Mean EMG activity in PM (top) while responding to rightward (first column) and leftward perturbations (second column) and in PD (bottom) while responding to rightward (first column) and leftward (second column) perturbations in the second experiment. The magenta, blue, and green traces represent the mean EMG activity measured when participants reached the left, center or right target, respectively. ***B***, Binned EMG activity before force onset in PM (top) and PD (bottom) for the leftward and rightward perturbation loads, for the trials that reached the central (left bin) and lateral (right bin) targets. ***C***, Group mean and SEM of the differences in forward velocities across participants between the center and lateral trials for trials with rightward (left) and leftward (right) perturbations. ***D***, Comparison of the forward velocity at force onset for the trials that reached the central (blue) and lateral (green or magenta) targets with a rightward or leftward perturbation load; *p* < 0.05 (*), *p* < 0.01 (**), *p* < 0.005 (***).

Interestingly, we observed a link between the state of the limb at perturbation onset (kinematics and EMG activity) and the outcome of the motor decision. Figure 5*A* represents the mean EMG activity recorded in PM (top) and PD (bottom) in presence of mechanical perturbations (rightward, first column and leftward second column) across participants for the different targets (magenta, left; blue, center; green, right) in the same values condition. No significant differences were observed in PM before force onset (−150 to 0 ms; Fig. 5*A*, gray rectangle) between the trials that reached the center target and the ones that reached the lateral targets (Fig. 5*B*, top) for both force directions (left: linear mixed model $\beta_{1}$ = −0.019, *t* = −1.76, *p* = 0.0782, $r^{2}$ = 0.62 and right: linear mixed model $\beta_{1}$ = 0.0054, *t* = 0.89, *p* = 0.3758, $r^{2}$ = 0.64). However, we observed an increase in the EMG activity of PD before perturbation onset for the trials that reached the center target compared with the ones that reached the lateral targets for both force directions (Fig. 5*B*, bottom, left, linear mixed model $\beta_{1}=0.022$, *t* = 3.78, *p* < 0.005, $r^{2}$ = 0.68; right, $\beta_{1}$ = −0.051, *t* = −4.804, *p* < 0.005, $r^{2}$ = 0.60). This increase in EMG activity for trials that ended at the central target was correlated with larger forward velocities at force onset. We reported in Figure 5*C* the differences in forward velocities between the center and the lateral trials for both perturbation directions. In presence of a leftward perturbation (Fig. 5*C*,*D*, right panels), we observed a larger forward velocity at force onset for trials that end up at the center target compared with those that reached the lateral target (linear mixed model: $\beta_{1}$ = −0.013, *t* = −3.347, *p* < 0.005, $r^{2}$ = 0.54). The same holds for trials with rightward perturbations (linear mixed model: $\beta_{1}$ = 0.040, *t* = 9.476, *p* < 0.005, $r^{2}$ = 0.57; Fig. 5*C*,*D*, left panels). Similar observations were reported in the different values conditions. Indeed, we observed an increase in EMG activities in both muscles for the trials that ended up at the center target compared with those that reached the lateral target (PM linear mixed model: $\beta_{1}$ = −0.051, *t* = −4.81, *p* < 0.005, $r^{2}$ = 0.60 and PD linear mixed model: $\beta_{1}$ = 0.022, *t* = 3.78, *p* < 0.005, $r^{2}$= 0.68). Moreover, some tendencies were observed in the forward speed for trials with rightward ( $\beta_{1}$= −0.011, *t* = −2.019, *p* = 0.0436, $r^{2}$ = 0.52) and leftward (linear mixed model $\beta_{1}$ = 0.012, *t* = 1.95, *p* = 0.0505, $r^{2}$ = 0.57). These results collected in the different values conditions have to be analyzed with caution because of the low number of trials that ended up at one of the two lateral targets (7.5% in the different values and 23.5% in the same values condition).

We also tested whether the reward condition (i.e., same values and different values) modified movement vigor by comparing the forward velocities and muscle activities at force onset between both reward conditions. We did not observe any difference in forward velocities between both reward conditions at perturbation onset as reported by mixed effect models (*t* = 0.60, *p* = 0.54, $r^{2}$ = 0.03). Similarly, we did not observe any differences in EMG activities averaged during the 50 ms preceding perturbation onset as revealed by mixed model analyses (PM: *t* = −0.3962, *p* = 0.69, $r^{2}$ = 0.28 and PD: *t* = 0.07, *p* = 0.93, $r^{2}$ = 0.06). The same observation holds for reaction times that did not show any modulation with the reward condition (linear mixed model, *p* > 0.05). This absence of correlation between the reward condition and movement vigor was interesting as it confirmed that we did not introduce any experimentally induced modulation of vigor in our paradigm. The differences in switching frequencies observed between the same and different values conditions are therefore attributable to the reward distribution and to vigor variability within both reward conditions.

This second experiment showed that humans take the rewards of the competing options into account to respond to perturbations and potentially change target goal during movement. More specifically, participants will tend to reduce their frequency of reaching toward targets that have a lower reward. We also showed that the state of the limb at perturbation onset modulated participants’ behavior. Indeed, higher feedbacks gains at perturbation onset were correlated with a higher probability to potentially change target goal during movement, conditioned by the occurrence of mechanical load that would push the limb toward lateral target.

### Effect of the preactivation of muscle on the motor decision

An outstanding question is when was the decision made to switch target. Did participants decide to change after the perturbation, or did they plan to change before movement? On the one hand, in this experiment as in previous reports (Nashed et al., 2014), changes in goal target depend on the occurrence and magnitude of the force so it is at least partially determined by sensory information collected during movement. On the other hand, the observation that the switch also depended on the baseline activity suggests that there could be an influence of the state of the limb from the beginning of the movement on the decision. We wanted to investigate this possibility in experiment 3. This experiment was specifically designed to investigate whether the preactivation of PD before movement onset could bias the frequency of target switches. Participants had to reach any of the three targets located at the same position as in experiment 2. All targets had the same reward in this experiment. During movement, mechanical perturbation loads could push participant’s hand orthogonally to the main reaching decision. During half of the trials, a leftward background load was applied to participant’s hand throughout movement evoking a background activation to counter the background load (see Materials and Methods). We assessed the effect of preactivation of PD by investigating the reach proportions to the lateral targets as a function of force intensity and background condition.

**Figure 6.**
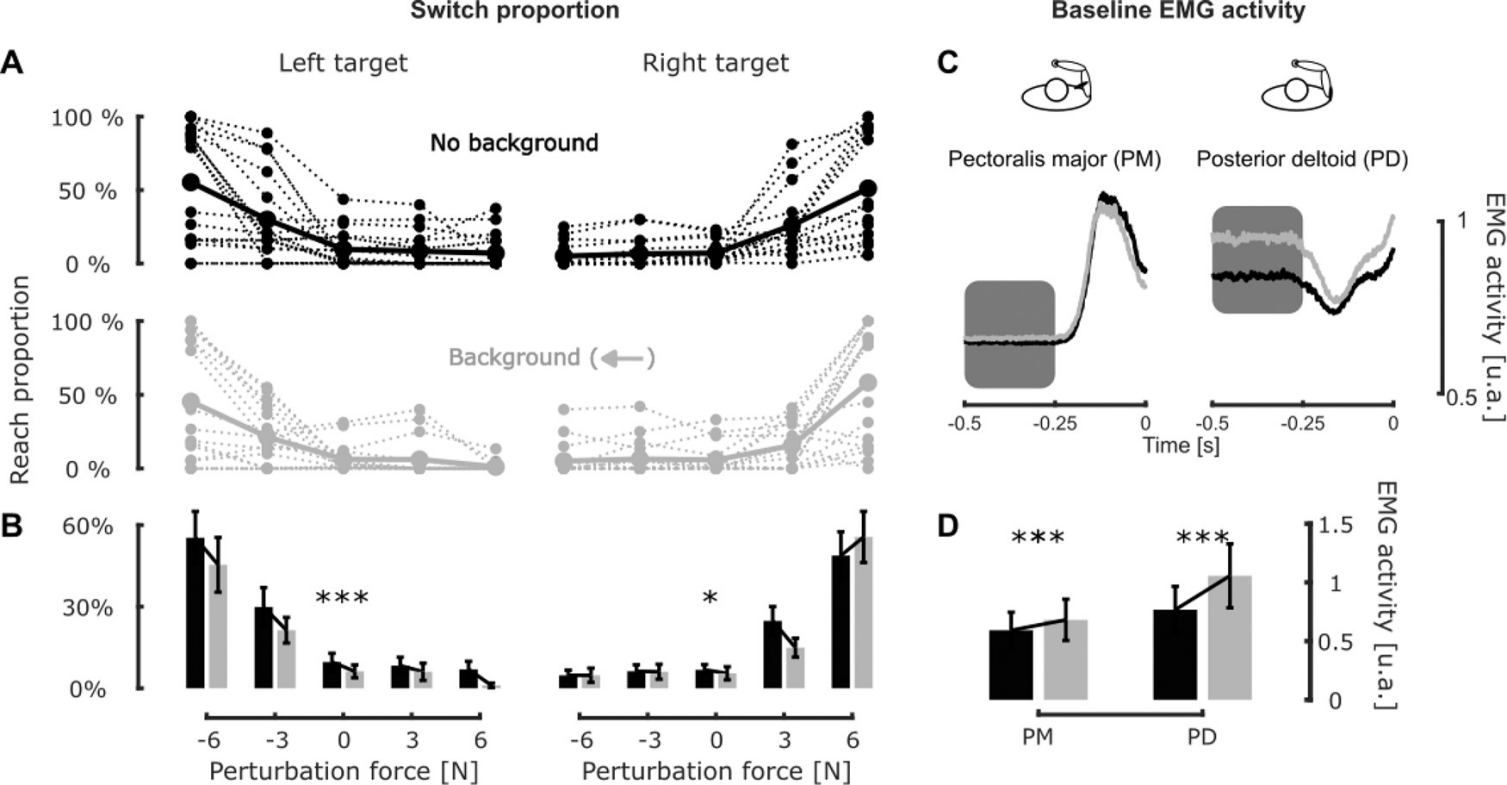
Experiment 3. ***A***, Group mean (full line) and individual means (dashed lines) of the reach proportion to the left and right targets for the conditions without (top row, black) and with (bottom row, gray) the leftward background load as a function of perturbation load. ***B***, Comparison of the reach proportion of the left and right targets (left and right columns, respectively) with (gray boxes) and without (black boxes) the leftward background force. ***C***, Group mean of the EMG activity in PM (left) and PD (right) before movement onset for trials without (black) and with (gray) a background load. The time axis is aligned with the perturbation load onset. ***D***, Comparison of the binned EMG activity between 500 and 300 ms (corresponding to the gray box in panel ***C***) before force onset in PM and PD for the conditions with and without background; *p* < 0.05 (*), *p* < 0.01 (**), *p* < 0.005 (***).

The application of a leftward background force induced an increase in both PM and PD baseline EMG activity (Fig. 6*C*). We found a significant effect of the background load in both muscles (main effect of the linear mixed model on both muscles: $\beta_{1}$ = −0.11, *t* = −6.73, *p* < 0.005, $r^{2}$ = 0.91) as represented in Figure 6*D*. Moreover, we also observed an interaction effect between the background load and the muscle: baseline activity in PD increased more than PM activity ( $\beta_{12}$= 0.20, *t* = 19.067, *p* < 0.005, $r^{2}$ = 0.91).

We found that the leftward background load modified the reach proportion to the left target for all kind of online mechanical loads. Figure 6*A* represents the reach proportions to the left and right targets (respectively, left and right column) as a function of the intensity of the perturbation load for the trial with (bottom) or without (top) background. In order to show the effect of the background load on the reach proportion to the lateral targets, we fitted a multilinear logistic regression (see Materials and Methods) that inferred the effect of perturbation and background load on the reached target. This multilinear logistic regression revealed a significant effect of the background and perturbations loads on both the left and right targets reaching proportion. Concerning the perturbation load, we observed an increase of the reach proportion to the left target with increasing leftward force ( $\beta_{1}$= −0.9223, *t* = −22.16, *p* < 0.005) and the mirror effect for the right target ( $\beta_{1}$ = 1.0204, *t* = 23.86, *p* < 0.005). The background load also had a significant effect on the reach proportion for these two targets. The reach proportion to the left target decreased when the background load was applied ( $\beta_{1}$ = −0.4611, *t* = −6.1759 and *p* < 0.005; Fig. 6B, left panel). Intuitively, an increase in force toward a target could bias the choice for that target but it was not the case. A slight decrease in reach proportion for the right target was also revealed by this regression ( $\beta_{1}$ = −0.1544, *t* = −1.9972, *p* = 0.0458; Fig. 6*B*, right panel). The intensity of this effect on the two lateral targets was compared using bootstrap resampling: this effect was larger for the left than for the right target. We generated 1000 bootstrap datasets from the original dataset used to fit the multilinear logistic regression and fitted the multilinear regression on each of these bootstrap datasets (generating that way estimates of $\beta_{1}$ for each resampled dataset). We extracted bootstrap estimates of the main effect of background on the target reached for both lateral targets and computed the difference between the left and right estimates. The mean value of this difference was 0.280 and the 95% confidence interval obtained from bootstrap resampling was [0.072, 0.503], which therefore indicates a non-zero difference. This result suggests a directional bias in the effect of the background load on the switching strategies: the application of a leftward background load hindered switches to the left target more than those to the right one.

*Post hoc* analyses performed on the individual reach proportion to lateral targets confirmed this asymmetry between left and right target (see Fig. 6*B*). We observed a significant decrease of the individual reach proportion to the left target induced by the background load across participants and force levels (one tailed Wilcoxon signed-rank test: *z* = 2.83, ranksum = 999.5, *p* < 0.005, *d* = 0.21). No similar effect was observed for the right target (Wilcoxon signed-rank test: *z* = 1.23, ranksum = 1015, *p* = 0.2154, *d* = 0.03).

An interesting question is whether this background force also modulated forward velocity. We address this question by using a linear mixed model to compare forward speed at force onset in the conditions with and without background load. No modulation of movement speed between these conditions was observed (linear mixed model $\beta_{1}$ = −0.009 ± 0.007, *t* = −1.2528, *p* = 0.210, $r^{2}$ = 0.44). This result is important as it discards the eventuality that the modulation of flexibility to switch to a new target goal was induced by movement velocity. Similarly, the reaction time was not modulated by the presence of the background force (linear mixed model, *p* > 0.05).

The results of this last experiment showed that the tendency to switch observed in experiment 2 depended on the biomechanical state of the limb. Importantly, the application of a background load in a direction reduces the tendency to switch in this direction in a larger amount than the tendency to switch in the opposite direction.

## Discussion

We conducted a series of experiments to investigate the impact of reward on feedback control strategies and rapid motor decisions by probing the impact of explicit target reward. In experiment 1, we demonstrated that target reward does not only increase movement vigor as reported in previous studies (Summerside et al., 2018; Yoon et al., 2018), but it also increases feedback responses and muscle activity. We observed that perturbation-related lateral hand deviations were smaller when participants reached toward a target associated with higher reward. Moreover, we also observed an increase in the baseline EMG activity as well as an increase in the EMG responses to perturbation loads with increasing reward. Altogether, these results suggest that the feedback gains used to perform movements scale with the value of the reward. In the second experiment, we reported that the reward distribution across the competing options has an influence on rapid motor decisions: participants were less prone to switch to a nearby target if it was associated with lower reward. In addition, an increase in feedback gains was detrimental to the ability to switch target during movement as we observed in experiments 2 and 3 that participants were also less likely to switch target during movement when the muscle activity was higher. The modulation in muscle activity introduced experimentally in experiment 3 induced a directional bias in the ability to switch target online, demonstrating a causal influence of muscle activity.

The increase in movement vigor and feedback gains associated with reward that we observed in experiment 1 was coherent with the selection of a robust control strategy (Crevecoeur et al., 2019; Bian et al., 2020). A robust controller consists in an alternative to stochastic optimal control (Todorov and Jordan, 2002) that has the property to consider unmodelled disturbances (Basar and Bernhard, 1991), which results in better responses to mechanical perturbations during movements. Reward is known to invigorate movements as revealed in saccadic eye movements where faster movements were observed toward higher monetary rewards (Manohar et al., 2015, 2017) or toward targets associated with higher implicit rewards (Xu-Wilson et al., 2009). Similar observations were made for upper limb reaching movements that exhibited higher peak velocities toward more rewarding targets (Sackaloo et al., 2015; Esteves et al., 2016; Summerside et al., 2018; Yoon et al., 2018). This was taken as evidence for reward-dependent selection of movement time (Shadmehr et al., 2010; Haith et al., 2012). It has recently been demonstrated that this increase in movement vigor was associated with higher muscle activity in presence of reward (Codol et al., 2020) which could be interpreted as a mechanism used to increase internal feedback gains to improve reward-related endpoint accuracy (Manohar et al., 2019). Here, we postulate that another mechanism is also at play: a higher reward produced a more robust strategy that revealed participants’ will to render their movements less sensitive to perturbations, thereby reducing the risk to miss the goal. In this framework the reduction in movement time results from the robustness of the control that impacts movement velocity through larger goal directed control gains.

In this framework, the modulation of the robustness of control has a clear limitation that we were able to establish empirically: a robust control strategy is meant to reject disturbances indistinguishably, thus in principle, it is clear that this strategy is not compatible with a flexible change in movement goal online, which requires a reduction in feedback response to let the perturbation redirect one’s hand toward the new goal.

Besides the property of the robust model to predict larger feedback gains, we measured here as in previous work that this strategy was associated with an increase in baseline co-activation (Crevecoeur et al., 2019) which potentially influences the gains of short-latency and long-latency responses to mechanical perturbations (Marsden et al., 1976; Bedingham and Tatton, 1984; Verrier, 1985; Matthews, 1986; Stein et al., 1995; Pruszynski et al., 2009). Considering this, the competition between robust control and flexible online decisions in the human motor system may depend in part of the fact that the robust controller recruits the peripheral motor apparatus (i.e., muscle state and reflex gains) to increase the overall feedback gains, thereby creating a competition between peripheral mechanisms engaged in control and more central decisional processes.

Moreover, our results demonstrate that the model based on distributed consensus of decision-making (Cisek, 2012) also applies to online motor decisions. This framework posits that decisions occur through an competition between the different options by integrating the motor costs incurred to each action (Cos et al., 2011; Shadmehr et al., 2016; Morel et al., 2017) and their respective outcome (Trommershäuser et al., 2003, 2008). We documented a concomitant influence of both the reward distribution across competing options and load magnitudes which highlights that these two factors are taken into consideration during online motor decision. In addition, we add to these factors that the state of the peripheral motor system, influenced by the selected control strategy and feedback gains, had an effect on online decision-making. Our findings are in line with previous work reporting an impact of the cost of each action (Nashed et al., 2014; Kurtzer et al., 2020; Michalski et al., 2020) and their associated outcome (Marti-Marca et al., 2020; Cos et al., 2021). These observations support that online motor decisions must result from distributed consensus between control strategies, feedback responses and rewards. Importantly, the present study investigated participants’ decision to switch to alternative targets during movement, all of which leading to successful movements, there were no good or bad choices as it is the case in a go-before-you-know paradigm (Chapman et al., 2010; Gallivan et al., 2016, 2017; Enachescu et al., 2021).

To conclude, our study highlights that multiple mechanisms underlie reward-dependent planning and control of movement. One the one hand, we suggest that there is a robust control strategy that involves peripheral circuits by means of increases in baseline activity and gain scaling of the feedback responses. This strategy associated with robust control is likely selected to reject perturbations and reduces the risk of missing the reward suggesting that there could be a cost incurred to reward. On the other hand, there exists a more flexible control strategy able to switch target during movement. It is conceivable that this second strategy, which requires some inhibition of muscle activity and response, is mediated by higher level inhibitory circuits and response modulation (Shadmehr and Krakauer, 2008; Scott, 2016). Both strategies integrate explicit target rewards and depend on the state of peripheral control loops.

An interesting open question is whether and how much individuals can modulate their strategy or whether the differences in strategy reflect individual traits. Indeed, individual differences have been shown in movement vigor (Reppert et al., 2018), and their possible effect on the modulation of feedback control is an exciting open question. Such ability is potentially central to understand planning and control in complex environments.

## Synthesis

Reviewing Editor: David Schoppik, New York University - Langone Medical Center Decisions are customarily a result of the Reviewing Editor and the peer reviewers coming together and discussing their recommendations until a consensus is reached. When revisions are invited, a fact-based synthesis statement explaining their decision and outlining what is needed to prepare a revision will be listed below. The following reviewer(s) agreed to reveal their identity: Thomas Reppert, Luc Selen.

In discussion with the two reviewers, four key points came up that need to be addressed. Briefly:

1. Add/assess reaction time data (also note the Enachescu paper (2021)).

2. Discuss effort expenditure as a contributing variable

3. Add/assess data on fatigue with either a longitudinal evaluation of behavior and/or reaction times.

4. Update the Introduction to better encompass the objectives of the experiments presented in the paper.

Below are the specific details from both reviewers, split by “major” and “minor” comments.

Major points:

1. The setup of Experiment 3 does not provide sufficient rebuttal to the possibility of a decision made prior to movement onset in Experiments 1 and 2.

a. One key confound that the authors mention is that of whether or not a decision is made by subjects prior to movement initiation. If this were true, then the observation that EMG activity at movement onset predicts choice could simply be explained by an early decision.

b. One key behavioral measure that is not mentioned in the manuscript, but that is of utmost importance, is reaction time. If the authors were to provide an assessment of reaction time, and its relation to the decision made by the subject, this would provide a more direct answer to the question of whether or not the decision-making process was complete by the time of movement start. This is particularly of note with respect to your discussion from Ln 525 onwards should maybe also include a very recent paper by Enachescu et al (Enachescu, V., Schrater, P., Schaal, S., & Christopoulos, V. (2021). Action planning and control under uncertainty emerge through a desirability-driven competition between parallel encoding motor plans. PLoS computational biology, 17(10), e1009429.) There, they evaluate reaction time for choice trials and they show that faster RTs are associated with ‘direct’ reaches to either of the two, whereas slow RTs are in an intermediate direction.

c. The magnitude and significance of the effect of the baseline force applied (Experiment 3) on movement choice was noticeably larger for choice of the left target relative to the right target. It is not clear why there would be a directional bias to the effect of baseline state on movement decision if such an effect is present.

d. If both the PM and PD muscles contribute to the initiation and execution of a successful movement, then it is unclear why early PD EMG activity predicts movement onset, but early PM activity does not.

2. One factor that is not mentioned in the manuscript is that of effort expenditure. The observation that reward influences movement kinematics and movement choice is clearly shown. For example, the authors show in Experiment 2 that reward biases movement choice (“different values” condition). However, the results of the “same values” condition also make it clear that effort plays a role in determining the outcome of the movement. As effort required to adjust the movement is increased, the probability of a corrected movement drops. The possibility of an effect of effort expenditure on movement decision should at least be acknowledged in this work.

3. The authors did not address the potential confound of trial number and subject fatigue. It is possible that certain results could be explained by muscle fatigue with repeated movements. This could lead to potential changes in both movement velocity and EMG activity at movement onset.

4. The primary objective(s) of the work are not sufficiently summarized. In the abstract and introduction, the authors highlight the effect of reward on movement kinematics and feedback control during the movement. However, the latter part of the paper (Figs. 5 and 6) primarily addresses the question of how contractile state of the limb affects choice. The introduction should provide a better connection between the primary objectives and the diverse findings of the work.

Minor points:

1. Order of presentation of the “background” and “no background” trials for Experiment 3 should have been counter-balanced across participants to remove the possibility of an order effect on the test condition.

2. Some of the levels of significance shown in plots do not match p-values presented in the text (e.g., Line 446 “p<0.005", yet in Fig. 6B significance shown at level ***).

3. The percentage of perturbation trials differed across the three experimental setups. It is not clear how a difference in the expectation of a trial with perturbation might influence the movement decision.

4. The authors mention an analysis of the relationship between reward condition and forward velocity for Experiment 2. It is unclear what is meant by “forward velocity” in this description. Is this average forward velocity of the movement?

5. No justification is provided for the 100-ms cutoff between long-latency and voluntary timeframe designations during the movement (Experiment 1).

6. Please describe your setup completely. Relying on EMG I assume you have airsled support? Also, it is unclear how the arm is positioned. You describe it, but I cannot work it out. And a 25cm reach, is that to full extension?

7. You use ‘home target’ and ‘goal target’ etcetera. Please use consistent names, e.g. ‘start location’, ‘target locations’.

8. Already on line 65-66 movement velocity and fb-gains are correlated. On Ln 302-304 you come back to this by comparing trials with similar peak velocity. This is a crucial analysis, but the description is rather limited.

9. Ln 343: Consider ‘lower rewards’.

10. Ln 565: somehting went wrong with the Carroll reference

11. Ln 727 ‘in the second experiment’ is superfluous.

Figure 2: Figure 2B, consider perhaps whether there a better way of plotting this (similar to ‘C’)?

Figure 3F Similarly to 2B, this was perceived as confusing.

Figure 6A, Why does the muscle activity have two ‘blips’. What is the grey box, what is the point of alignment (’0’)?

## Author Response

We would like to thank the Reviewers and the Editor for their in-depth assessment of our work and for raising the important points below. These helped us to significantly improve the paper. Please find below a point-to-point response to the comments that were made along with references to the modifications in the text.

Synthesis Statement for Author (Required):

In discussion with the two reviewers, four key points came up that need to be addressed. Briefly:

1. Add/assess reaction time data (also note the Enachescu paper (2021)).

2. Discuss effort expenditure as a contributing variable

3. Add/assess data on fatigue with either a longitudinal evaluation of behavior and/or reaction times.

4. Update the Introduction to better encompass the objectives of the experiments presented in the paper.

Below are the specific details from both reviewers, split by “major” and “minor” comments.

Major points:

1. The setup of Experiment 3 does not provide sufficient rebuttal to the possibility of a decision made prior to movement onset in Experiments 1 and 2.

a. One key confound that the authors mention is that of whether or not a decision is made by subjects prior to movement initiation. If this were true, then the observation that EMG activity at movement onset predicts choice could simply be explained by an early decision.

Importantly, we agree that a form of decision was taken by participants prior to movement onset, but it was not the decision to switch, instead it was a decision to switch conditional on the occurrence of a perturbation (see the corresponding analyses on lines 368-373 and Figure 4a). The target that will eventually be reached could not be predicted from the EMG activity since the magnitude and direction of the mechanical perturbation was unknown. However, this modulation of the EMG activity prior to perturbation onset allows predicting whether participants were more or less likely to switch to an alternative target. Hence the decision that is potentially taken beforehand is a strategy (e.g. counter the load or let your hand go), but it was not a decision associated with an alternative target. The summary of the Results section describing this was revised (lines 428-431).

To address this comment, we performed an additional analysis of the EMG activity in Experiment 2 and focused on an earlier time epoch (from -150ms to -100ms). We observed larger EMG activity in PD for the trials that reached the center target compared to the ones that reached the lateral targets for both force directions (left: linear mixed model β_1=-0.038, p<0.005, t=-3.01 and r^2=0.58 and right: linear mixed model β_1=0.011, p=0.014, t=2.44 and r^2=0.75). Such an increase in EMG activity was absent in PM for both force directions (left: linear mixed model β_1=0.006, p=0.11, t=1.56 and r^2=0.72 and right: linear mixed model β_1=-0.028, p=0.048, t=-1.97 and r^2=0.60).

b. One key behavioral measure that is not mentioned in the manuscript, but that is of utmost importance, is reaction time. If the authors were to provide an assessment of reaction time, and its relation to the decision made by the subject, this would provide a more direct answer to the question of whether or not the decision-making process was complete by the time of movement start. This is particularly of note with respect to your discussion from Ln 525 onwards should maybe also include a very recent paper by Enachescu et al (Enachescu, V., Schrater, P., Schaal, S., & Christopoulos, V. (2021). Action planning and control under uncertainty emerge through a desirability-driven competition between parallel encoding motor plans. PLoS computational biology, 17(10), e1009429.) There, they evaluate reaction time for choice trials and they show that faster RTs are associated with ‘direct’ reaches to either of the two, whereas slow RTs are in an intermediate direction.

We thank the reviewers for this interesting suggestion. We investigated the reaction times in all three experiments to explore a potential correlation with the behavioral parameters that we reported: in Experiment 1, we investigated whether target reward modulated the reaction time, while in Experiments 2 and 3, we investigated whether the reward distribution, the background force of the target reached at the end of the trial modulated the reaction time. We did not observe any modulation of reaction time for any of these tests (p>0.05 for each test). This information was added to Results section of the revised manuscript (lines 262-265, 419-420, and 491-492).

The fact that reaction times were statistically similar likely resulted from the way we designed the experimental paradigms (see lines 108-109 and 148). Indeed, there was no constraint on the reaction time and participants could initiate their movement whenever they wanted after go cue. Consequently, the reaction times we measured were longer than those reported in other studies (we measured RT between 350 and 500ms across experiments). Importantly, Experiments 2 and 3 implement the change-of-mind paradigm (Cos et al., 2021; Martí-Marca et al., 2020; Michalski et al., 2020) in which the decision to switch to an alternative target occurs during movement. Since we probed conditional decisions that happened during movement, reaction times have less impact than in the go before you know paradigm (Chapman et al., 2010; Enachescu et al., 2021; Gallivan et al., 2016), where participants are instructed to initiate movement before knowing which option is the correct one. This difference was added to the Discussion section of the revised manuscript (lines 547-562).

c. The magnitude and significance of the effect of the baseline force applied (Experiment 3) on movement choice was noticeably larger for choice of the left target relative to the right target. It is not clear why there would be a directional bias to the effect of baseline state on movement decision if such an effect is present.

This is a very important point that needed to be clarified. In Experiment 2, we demonstrated a correlation between the muscle activity at perturbation onset and the outcome of the decision process triggered by that perturbation: higher muscle activities were correlated with a smaller proportion of switches to the lateral targets (lines 386-412). Elaborating on that, we hypothesized that higher muscle activity at perturbation onset reduced participants’ propensity to switch target during movement. We therefore decided to directionally bias the background activity by introducing a constant background load throughout preparation and execution phases. This induced an asymmetry in the muscle activity (higher increase in PD than in PM) that we could correlate with an asymmetric reduction of the reach frequency to the lateral targets (higher decrease for the leftward target).

The mechanism whereby this directional bias on movement choice happens is not fully resolved but we put forward the known relationship between higher background activity and a transient increase in short and long-latency stretch responses (gain-scaling : lines 538-546).

d. If both the PM and PD muscles contribute to the initiation and execution of a successful movement, then it is unclear why early PD EMG activity predicts movement onset, but early PM activity does not.

This is an interesting comment and we agree that it needed to be clarified. The reason why we investigated the EMG activities of PM and PD is because, in previous studies (Crevecoeur et al., 2019; De Comite et al., 2021), it was reported that these muscles produce stereotypical stretch responses to rightward and leftward perturbations similar to those that we were planning to use in the present study. The contribution of both muscles to movement differ because of the specific configuration of participant’s arm during movement (see comment in the Minor points). During the early phases of movements, the contribution of PD is larger than that of PM. However, in presence of lateral mechanical perturbation, each muscle is recruited by stretch responses in presence of leftward (PD) or rightward (PM) perturbations. This point and the 6th minor comment were clarified in the Methods section of the revised manuscript (lines 95-96 and lines 188-191).

2. One factor that is not mentioned in the manuscript is that of effort expenditure. The observation that reward influences movement kinematics and movement choice is clearly shown. For example, the authors show in Experiment 2 that reward biases movement choice (“different values” condition). However, the results of the “same values” condition also make it clear that effort plays a role in determining the outcome of the movement. As effort required to adjust the movement is increased, the probability of a corrected movement drops. The possibility of an effect of effort expenditure on movement decision should at least be acknowledged in this work.

This is true that effort expenditure also influences kinematics and movement choice and this prediction is borne out in the data we collected in the “same values” condition (lines 547-562 for the part of the discussion that has been modified to highlight this aspect in more details). Indeed, the frequency of switch depended on the load magnitude, thus also on the actual hand displacement following the perturbation. Since targets had the same reward, changes in switch frequencies must have been related to movement kinematics and associated efforts. This result indicates that participants also take future effort expenditure into account during this decision process as it was shown in past studies (Cos et al., 2011; Morel et al., 2017).

3. The authors did not address the potential confound of trial number and subject fatigue. It is possible that certain results could be explained by muscle fatigue with repeated movements. This could lead to potential changes in both movement velocity and EMG activity at movement onset.

To address the reviewers’ concern, we investigated the impact of muscle fatigue in our data by looking at the evolution of peak-velocity across blocks. In all experiments, we observe a very small decrease in peak velocity across blocks (<1% of reduction). Moreover, we designed the experimental protocols such that the impact of fatigue was minimized. Indeed, the trials were grouped in blocks of 72 (Expe. 1) or 80 (Expe. 2-3) trials separated by self-timed pauses that allowed participants to rest between blocks (these self-timed pauses lasted on average between 2 and 5 minutes - added in the revised version of the manuscript on lines 119-120, 156-157 and 175-176). In addition to these pauses, participants were allowed to take short breaks within a block between two trials as it did not interfere with the task.

We cannot formally rule out the impact of fatigue in our paradigms but we carefully designed the experiments to minimize its impact on the measured variables. The coherence of the kinematic behavior across blocks, as characterized by the peak-velocity, and the presence of condition specific modulation of behavior in all three experiments (consider for instance the asymmetry in the reduction of switching frequencies reported in Experiment 3) suggest that the impact of fatigue in our results is negligible.

4. The primary objective(s) of the work are not sufficiently summarized. In the abstract and introduction, the authors highlight the effect of reward on movement kinematics and feedback control during the movement. However, the latter part of the paper (Figs. 5 and 6) primarily addresses the question of how contractile state of the limb affects choice. The introduction should provide a better connection between the primary objectives and the diverse findings of the work.

The link between the first experiment and the last two ones has been strengthened in the introduction (lines 61-69). The role of Experiment 1 was to reveal the modulation of the robustness of control policies used by humans to perform reaching movements by leveraging the reward associated with the task. By doing so, we demonstrated that more robust control strategies are correlated with higher feedback gains which could be detrimental to one’s ability to switch target during movement. In Experiment 2 and 3 we have shown that indeed one’s capacity to change target during movement was impaired when higher feedback gains were selected. In all we demonstrated that the selection of robust control strategies characterized by higher feedback gains impacted rapid motor decisions.

Minor points:

1. Order of presentation of the “background” and “no background” trials for Experiment 3 should have been counter-balanced across participants to remove the possibility of an order effect on the test condition.

We agree that a counterbalanced design would have been ideal. However, the asymmetry that we reported in the reduction of switch frequency to the lateral targets (see lines 455-485) was clearly linked to the direction of the background force, whereas a potential effect of fatigue would have resulted in a symmetrical reduction in switching frequencies. It is also unclear whether potential presence of fatigue could have impacted our results because the switching frequency increased in the direction of the muscle that was not recruited by the background force.

2. Some of the levels of significance shown in plots do not match p-values presented in the text (e.g., Line 446 “p<0.005", yet in Fig. 6B significance shown at level ***).

This was corrected. We defined the characters for the representation of the p-values on lines 241-242 and added it in all the figure legends to make it clearer.

3. The percentage of perturbation trials differed across the three experimental setups. It is not clear how a difference in the expectation of a trial with perturbation might influence the movement decision.

Although the frequency differed across experiments, we made sure that the proportion was kept constant within blocks of each experiments, and that the trials were randomized such that participants could not predict the occurrence or direction of a mechanical perturbation. The data presented here do not allow to investigate a potential influence of the expectation of perturbation could have on the motor decision. Based on a previous report (Crevecoeur et al., 2019), one can expect that a higher frequency leads to a more robust strategy and hence overall lower proportion of switches. We believe that this is beyond the scope of our study and represents an interesting question for future work.

4. The authors mention an analysis of the relationship between reward condition and forward velocity for Experiment 2. It is unclear what is meant by “forward velocity” in this description. Is this average forward velocity of the movement?

The forward velocity mentioned here was the velocity component aligned with the main movement axis. This has been defined on lines 256-257.

5. No justification is provided for the 100-ms cutoff between long-latency and voluntary timeframe designations during the movement (Experiment 1).

We selected this cutoff value as it is a standard value to separate long-latency and early voluntary responses (Pruszynski et al., 2008; Pruszynski & Scott, 2010). This has been added to the methods section of the edited version of the manuscript (lines 198-200).

6. Please describe your setup completely. Relying on EMG I assume you have airsled support? Also, it is unclear how the arm is positioned. You describe it, but I cannot work it out. And a 25cm reach, is that to full extension?

There was no airsled support, in fact participants arm was not constrained. When their hand was located within the start location, the configuration was roughly aligned with the sagittal plane and their elbow formed an angle of about 90{degree sign} (the start location was aligned with their right arm). In the unperturbed case and if they were reaching to the central targets of Expe. 2 and 3, their movement was characterized by a shoulder abduction and an elbow extension. Their arm was never in full extension throughout the trials. The description of participants’ position has been edited in the manuscript (lines 94-96).

7. You use ‘home target’ and ‘goal target’ etcetera. Please use consistent names, e.g. ‘start location’, ‘target locations’.

We thank the Reviewers for pointing this out and we replaced the term “home target” by “start location” throughout the manuscript.

8. Already on line 65-66 movement velocity and fb-gains are correlated. On Ln 302-304 you come back to this by comparing trials with similar peak velocity. This is a crucial analysis, but the description is rather limited.

The importance of the analyses on the trials with similar velocity peaks has been stressed out in the revised version of the manuscript (see lines 316-318).

9. Ln 343: Consider ‘lower rewards’.

This has been changed

10. Ln 565: something went wrong with the Carroll reference

This has been fixed

11. Ln 727 ‘in the second experiment’ is superfluous.

This has been removed

Figure 2: Figure 2B, consider perhaps whether there a better way of plotting this (similar to ‘C’)?

Colors have been added to the legend to clarify that panel. It could indeed be interesting to represent the modulation of velocity similarly to what was done in panel C but the range of the full velocity trace masked the modulation across conditions, which is why we decided to represent the difference instead.

Figure 3F Similarly to 2B, this was perceived as confusing.

Colors have been added to the legend to clarify that panel. Same remark as above.

Figure 6A, Why does the muscle activity have two ‘blips’. What is the grey box, what is the point of alignment (‘0’)?

The blips correspond to the initiation of movement while the grey box represents the time bin in which the EMG has been averaged for panel D. The point of alignment is the onset of the mechanical perturbation. All these points have been clarified in the figure caption (lines 773-774).

References

Chapman, C. S., Gallivan, J. P., Wood, D. K., Milne, J. L., Culham, J. C., & Goodale, M. A. (2010). Reaching for the unknown : Multiple target encoding and real-time decision-making in a rapid reach task. Cognition, 116(2), 168‑176. https://doi.org/10.1016/j.cognition.2010.04.008

Cos, I., Bélanger, N., & Cisek, P. (2011). The influence of predicted arm biomechanics on decision making. Journal of Neurophysiology, 105(6), 3022‑3033. https://doi.org/10.1152/jn.00975.2010.

Cos, I., Pezzulo, G., & Cisek, P. (2021). Changes of mind after movement onset : A motor-state dependent decision-making process. eNeuro, 8(6), ENEURO.0174.

Crevecoeur, F., Scott, S. H., & Cluff, T. (2019). Robust Control in Human Reaching Movements : A Model-Free Strategy to Compensate for Unpredictable Disturbances. The Journal of neuroscience : the official journal of the Society for Neuroscience, 39(41), 8135‑8148. https://doi.org/10.1523/JNEUROSCI.0770-19.2019

De Comite, A., Crevecoeur, F., & Lefèvre, P. (2021). Online modification of goal-directed control in human reaching movements. Journal of Neurophysiology, 125(5), 1883‑1898.

Enachescu, V., Schrater, P., Schaal, S., & Christopoulos, V. (2021). Action planning and control under uncertainty emerge through a desirability-driven competition between parallel encoding motor plans. PLoS Computational Biology, 17(10), e1009429.

Gallivan, J. P., Logan, L., Wolpert, D. M., & Flanagan, J. R. (2016). Parallel specification of competing sensorimotor control policies for alternative action options. Nature Neuroscience, 19(2), 320‑326. https://doi.org/10.1038/nn.4214

Martí-Marca, A., Deco, G., & Cos, I. (2020). Visual-reward driven changes of movement during action execution. Scientific Reports, 10(1), 1‑12. https://doi.org/10.1101/656330

Michalski, J., Green, A. M., & Cisek, P. (2020). Reaching decisions during ongoing movements. Journal of Neurophysiology, 123(3), 1090‑1102. https://doi.org/10.1152/jn.00613.2019

Morel, P., Ulbrich, P., & Gail, A. (2017). What makes a reach movement effortful ? Physical effort discounting supports common minimization principles in decision making and motor control. PLOS Biology, 15(6), 1‑23.

Pruszynski, J. A., Kurtzer, I., & Scott, S. H. (2008). Rapid Motor Responses Are Appropriately Tuned to the Metrics of a Visuospatial Task. Journal of Neurophysiology, 100(1), 224‑238. https://doi.org/10.1152/jn.90262.2008

Pruszynski, J. A., & Scott, S. H. (2010). Optimal feedback control and the long-latency stretch reflex. Experimental Brain Research, 218, 341‑359.
